# Exploration of Molecular Network Variations in Different Subtypes of Human Non-functional Pituitary Adenomas

**DOI:** 10.3389/fendo.2016.00013

**Published:** 2016-02-10

**Authors:** Xianquan Zhan, Ying Long

**Affiliations:** ^1^Key Laboratory of Cancer Proteomics of Chinese Ministry of Health, Xiangya Hospital, Central South University, Changsha, China; ^2^Hunan Engineering Laboratory for Structural Biology and Drug Design, Xiangya Hospital, Central South University, Changsha, China; ^3^State Local Joint Engineering Laboratory for Anticancer Drugs, Xiangya Hospital, Central South University, Changsha, China; ^4^The State Key Laboratory of Medical Genetics, Central South University, Changsha, China

**Keywords:** non-functional pituitary adenoma, proteomics, omics data, systems biology, molecular network

Pituitary adenoma is a common disease occurring in pituitary that is a central regulatory organ in endocrine system and is clinically categorized as functional and non-functional pituitary adenomas (FPA and NFPA) (1–4). FPA commonly includes adrenocorticotropic hormone (ACTH)-cell adenoma, thyrotropin-stimulating hormone (TSH)-cell adenoma, growth hormone (GH)-cell adenoma, and prolactin (PRL)-cell adenoma (5). Compared to FPA, NFPA is a very challenging clinical problem due to no any increase of corresponding serum hormone, which results in difficulty in early-stage diagnosis and therapy of an NFPA (1, 2). Moreover, high-degree heterogeneity occurs in NFPAs, which is present as different subtypes of cell origins and hormones expressed in a tumor tissue; for example, intact gonadotroph with luteinizing hormone (LH)/follicle-stimulating hormone (FSH)-positive (40–79%), null cell without hormone expression (17%), oncocytoma without hormone expression (6%), silent corticotroph with ACTH-positive (8%), and silent somatotroph with GH-positive (3%). The intact gonadotroph is subdivided into LH^+^, FSH^+^, and LH/FSH^+^ positive NFPAs (5, 6). The very interesting thing is that the silent hormone expressed in an NFPA tissue associates tumor biological behaviors, such as invasive characteristics (7, 8); for example, a clinically silent corticotroph tumor of a pituitary shows symptomatic cystic degeneration (7), and estrogen receptors and slug contribute to development of invasiveness of an NFPA (8). In addition, NFPA is a very complex whole-body disease with multiple molecule dynamic alterations in the levels of genome, transcriptome, proteome, and metabolome, and those different levels of multiple molecules constitute three-dimensional spatial interactome to exert their biological roles in an NFPA biological system from an angle of multiparameter systematic strategy (9–12). The documented omics data demonstrate obvious variations in transcriptome and proteome between NFPAs and controls (6), between invasive and non-invasive NFPAs (13, 14), and among four hormone-expressed NFPA subtypes (1). Also, post-transcriptional splicing and numerous post-translational modifications (PTMs) contribute to the complexities of transcriptome and proteome and cause the interactome more complex and more dynamic (15); for example, the literature demonstrates the splicing variants/isoforms of hormones, such as GH and prolactin, and PTMs, such as tyrosine nitration and phosphorylation that occur in NFPAs (6, 16, 17). One should understand the interactome in three levels including alterations in single molecule, molecule profile, and molecular network profile in an NFPA. Molecular network is the carrier to recognize the interactome from a view point of systematic strategy. More attentions have been paid to molecular network of NFPA because it changes the single-one parameter model to multiparameter systematic strategy model, which meets the reality of complicated pathophysiological processes of an NFPA (9–11). Therefore, recognition of molecular network variations would benefit clarification of molecular mechanisms and discovery of reliable biomarkers and therapeutic targets of an NFPA toward personalized medicine (18) and precision medicine (19) practices.

In our studies on NFPA, two-dimensional gel electrophoresis (2DGE)-based quantitative proteomics, PTM-proteomics, bioinformatics, and pathway network-based systems biology were used to investigate the variations in proteome and protein molecular networks of an NFPA (1, 6, 14, 16, 20). (i) For NFPAs compared to pituitary controls (6, 16, 20), a set of 50 differentially expressed proteins (DEPs), 9 nitroproteins, and 3 nitroprotein–protein complexes were identified (6, 16). Pathway network analysis revealed four important pathway network systems that operate in an NFPA, including mitochondrial dysfunction, oxidative stress, cell-cycle dysregulation, and MAPK-signaling abnormality (20). (ii) For invasive relative to non-invasive NFPAs (14), a set of 57 DEPs were identified. Pathway network analysis revealed eight important pathway networks that associate the invasive characteristics of invasive NFPAs, including mitochondrial dysfunction, oxidative stress, MAPK-signaling abnormality, proteolysis abnormality, CDK5 signaling abnormality, ketogenesis and ketolysis, TR/RXR activation, and amyloid processing. (iii) For comparison of four hormone-expressed subtypes of NFPAs (NF^−^, LH^+^, FSH^+^, and LH/FSH^+^; NF− means NFPA that had negative immunohistochemical stains for ACTH, FSH, GH, LH, prolactin, and TSH) versus pituitary controls (Con) (1), a total of 76 DEPs was identified, including 59 DEPs in NF vs. Con, 65 DEPs in LH vs. Con, 63 DEPs in FSH vs. Con, and 55 DEPs in LH/FSH vs. Con. Overlapping and pathway network analyses revealed a set of DEPs and pathway networks that are common and specific to each NFPA subtype; of them, four important common pathway systems were MAPK-signaling abnormality, oxidative stress, mitochondrial dysfunction, and cell-cycle dysregulation. However, those four common pathway network systems were significantly different among four NFPA subtypes with variations in three different aspects, including different protein expression levels of most of protein nodes, different protein profiles, and different pathway network profiles (1, 21). With comprehensive consideration of three sets of quantitative proteomics data-based pathway network analyses between (a) NFPAs versus controls, (b) invasive versus non-invasive NFPAs, and (c) four hormone-expressed NFPA subtypes, some interesting results were found that (i) three pathway network systems including mitochondrial dysfunction, oxidative stress, and MAPK-signaling abnormality were identified from three sets of analyses (a)–(c), (ii) cell-cycle dysregulation was identified from two sets of analyses (a) and (c) but not (b), and (iii) the components (expression level of each protein node and protein profile) of each pathway network system were different among three sets of analyses (a)–(c). Those data clearly indicate the common and specific molecular network variations in NFPAs and among different subtypes of NFPAs.

Those data provide a preliminary view of molecular network variations in NFPAs and its subtypes. However, one should realize the limitations of those mentioned studies and the directions of further studies: (i) those molecular networks derived from 2DGE quantitative proteomics data were constructed with bioinformatics and systems biology. The biological relevance of each molecular network in the pathophysiological processes of an NFPA should be further studied with *in vitro* and *in vivo* experiments. (ii) 2DGE quantitative proteomics has obvious limitations in maximizing the coverage of an NFPA proteome. The 2DGE with an 18-cm pH3-10 NL IPG strip and 12% SDS-PAGE isolation in mentioned studies detected proteins within a range of Mr 5–150 kDa and pH 4–8 (5, 22). Multi-dimensional liquid chromatography (MDLC) in combination with tandem mass spectrometry (MS/MS) is needed to maximize the coverage of NFPA proteome for molecular network analysis (22). (iii) Protein variants/isoforms and PTMs are the important aspects in a proteome and associate many important physiological and pathological processes (15). Barely studies are found to explore globally and systematically protein variants/isoforms and PTMs in an NFPA and how those protein variants/isoforms and PTMs affect the molecular network systems in an NFPA, even though several PTMs, such as tyrosine nitration (16, 23) and phosphorylation (17, 24), and protein variants/isoforms such as GH isoforms and PRL isoforms (6, 25) were preliminarily investigated in NFPAs. (iv) Transcriptomics data between NFPAs and controls (6) and invasive and non-invasive NFPAs (13) have been documented. However, transcriptomics data-based molecular network analysis has not been systematically carried out in those studies (6, 13). An integrated molecular network based on omics data including genomic, transcriptomic, proteomic, peptidomic, and metabolomic data would have more important biological significance in the clarification of molecular mechanisms and discovery of reliable biomarkers for an NFPA patient (10). (v) NFPA is highly heterogeneous tumor. The documented molecular network variation studies between NFPAs versus controls (6, 20), invasive and non-invasive NFPAs (14), and four NFPA subtypes (NF^−^, LH^+^, FSH^+^, and LH/FSH^+^) subtypes (1) should be extended to other NFPA subtypes, such as silent ACTH- and GH-positive NFPAs, and different FPA subtypes, such as ACTH-, TSH-, GH-, and PRL-cell FPAs (5).

In summary, NFPA has high-degree heterogeneity and difficulty in its early-stage diagnosis and therapy. From the angle of systems biology strategy, exploration of variations in molecular networks that operate in an NFPA will be an important approach to the in-depth understanding of molecular mechanisms and discover effective and reliable biomarkers and therapeutic targets for personalized and precision medicine practices in an NFPA patient.

## Author Contributions

XZ conceived of the concept and idea of this article, collected references, wrote, and revised the entire manuscript, was responsible for the corresponding work of the manuscript, and trained YL regarding the concepts of molecular networks and systems biology in cancer. YL participated in the collection of references, revision, and formatting manuscript. All authors read and approved the final manuscript.

## Conflict of Interest Statement

The authors declare that the research was conducted in the absence of any commercial or financial relationships that could be construed as a potential conflict of interest.
